# Fluid Ability (Gf) and Complex Problem Solving (CPS)

**DOI:** 10.3390/jintelligence5030028

**Published:** 2017-07-13

**Authors:** Patrick Kyllonen, Cristina Anguiano Carrasco, Harrison J. Kell

**Affiliations:** Academic to Career Research Center, Research & Development, Educational Testing Service, Princeton, NJ 08541, USA; canguianocarrasc@ets.org (C.A.C.); hkell@ets.org (H.J.K.)

**Keywords:** complex problem solving, general fluid ability, O*NET, minimal complexity systems, abilities, domain knowledge, Program for International Student Assessment (PISA)

## Abstract

Complex problem solving (CPS) has emerged over the past several decades as an important construct in education and in the workforce. We examine the relationship between CPS and general fluid ability (Gf) both conceptually and empirically. A review of definitions of the two factors, prototypical tasks, and the information processing analyses of performance on those tasks suggest considerable conceptual overlap. We review three definitions of CPS: a general definition emerging from the human problem solving literature; a more specialized definition from the “German School” emphasizing performance in many-variable microworlds, with high domain-knowledge requirements; and a third definition based on performance in Minimal Complex Systems (MCS), with fewer variables and reduced knowledge requirements. We find a correlation of 0.86 between expert ratings of the importance of CPS and Gf across 691 occupations in the O*NET database. We find evidence that employers value both Gf and CPS skills, but CPS skills more highly, even after controlling for the importance of domain knowledge. We suggest that this may be due to CPS requiring not just cognitive ability but additionally skill in applying that ability in domains. We suggest that a fruitful future direction is to explore the importance of domain knowledge in CPS.

## 1. Introduction

Complex problem solving (CPS) has emerged over the past several decades as an important construct in cognitive psychology, and is viewed as an important applied skill in education and in the workforce [1,2]. In education, measures of problem solving or CPS have been included in several cycles of the Program for International of Student Assessment (PISA) [3,4,5]. In the workforce, “Complex Problem Solving” is one of the skills the USA Department of Labor [6] routinely evaluates for its importance in occupations for the O*NET database, and “Problem Solving” is a skill that is routinely asked about in employer surveys on skills required for the workforce [7]. At the same time, there is a lack of clarity and perhaps agreement on what exactly CPS is. For example, CPS has been criticized as an area of cognitive science that lacks a good definition, one that might serve to classify tasks as indicators of complex problem solving; this lack may have contributed to a lack of progress on the construct [8]. 

The ostensibly related construct of general fluid ability (Gf), defined as “the capacity to solve novel, complex problems, using operations such as inductive and deductive reasoning, concept formation, and classification” ([9], p. 423) also is an important one, has been shown to be predictive of success in education and the workforce, and has a long history of use in cognitive psychology, particularly differential (individual differences) psychology. In contrast to CPS, Gf arguably can be defined sufficiently for classifying tasks; at the very least, there is a large body of literature supporting such a classification [10]. 

A natural question is what is the relationship between Gf and CPS? This question has been the subject of debate over the past several years [11,12,13,14,15,16,17]. In this paper, we demonstrate that there is a strong relationship between fluid ability (Gf) and complex problem solving (CPS) skill, both conceptually and empirically. Conceptually, we show the connection by comparing definitions through a review of the two constructs. Empirically, we examine the correlations between ratings of Gf and CPS importance for several hundred occupations in the U.S. Department of Labor’s O*NET database. We believe that this analysis complements other approaches to the question of the relationship between CPS and Gf, which have examined the correlations between performance on CPS tests and performance on Gf tests (e.g., [15]). 

It is useful to appreciate the relationship between CPS and Gf for both theoretical and applied purposes. Theoretically, it is useful to understand CPS in relation to Gf to get a better understanding of CPS such as the potential role of working memory in CPS task performance, as has been found in Gf task performance (e.g., [18,19]), developmental and educational effects [20,21], and the worldwide secular trend [22,23,24]. Practically, it is useful to be able to predict CPS task performance from Gf measures. Given that CPS measures have been administered in several international student achievement and adult knowledge surveys [4,5,25], it is useful to understand how to interpret the findings.

We propose that there are two ways to view the relationship between Gf and CPS, depending on how tightly or loosely tasks are defined as CPS tasks:

1. Under a narrow, tight definition of CPS, in which tasks are classified as CPS tasks by common features and correlations in performance of them (i.e., reflective measures [26]), fluid ability can be viewed as the more general construct, with complex problem solving (CPS) as a task type or lower order construct that largely can be accounted for by fluid ability. As is the case with other lower-order constructs, such as quantitative, deductive, or inductive reasoning, this relationship does not preclude CPS from having unique features, such as a dynamic character and time sensitivity, in addition to features that overlap with other fluid ability factors, such as requiring inductive or deductive reasoning. Note that in the differential psychology literature, abilities are typically defined at three orders (or strata) of generality [10,27]: at the top (third) order, there is a general factor influencing performance on any cognitive task [28]; at the second order there are broad group factors, such as fluid, crystallized, and spatial ability; and at the first order there are narrower factors pertaining to types of cognitive processing activities, such as deductive reasoning, or inductive reasoning (the g-VPR model [29] also is based on a hierarchical arrangement of factors varying in generality). It is here that we would place a narrowly defined CPS—at the first order, within the span of fluid ability tasks, alongside inductive reasoning tasks (such as progressive matrices), or deductive reasoning tasks (such as three-term series tasks).
2. Under a broader definition of CPS, one that classifies a task as a CPS task based on meeting a set of criteria, whether or not the resulting set of tasks are correlated with one another, there may be alternative characterizations of the meaning of the CPS construct, such as treating it as a formative latent variable construct; that is, one defined by formative or cause indicators [26]. As such, fluid ability can be seen as an important and strong predictor of success on CPS tasks, with the strength of the relation varying depending on the particular CPS task.

Our approach in this paper is first to clarify definitions of CPS and fluid ability in order to observe common and distinctive features in those definitions. We also review the distinction between formative and reflective latent variable models: Whereas fluid ability emerged as a reflective latent variable model, through factor analysis, CPS emerged at least partly as a defined construct which is characteristic of constructs associated with formative latent variable models [30]. Failing to acknowledge the conceptual differences between the types of constructs can result in confusions in the literature concerning how to define, identify, and compare constructs [31].

It is useful to examine the relationship between CPS and fluid ability empirically. First, we show that in several PISA surveys, CPS as measured by various tasks is highly related to other PISA measures, including quantitative reasoning. We also note that PISA largely measures general cognitive ability [32], which is identical with fluid ability [33,34], or very similar [9,35]. Second, we show a strong relationship between fluid ability and CPS importance ratings at the occupation level, based on O*NET data from the USA Department of Labor [6,36]. Occupations that are judged to require high levels of fluid ability are also ones that are judged to invoke CPS. Both CPS and fluid ability are also correlated with occupational earnings and occupational zone, an index of overall occupational complexity and educational requirements. We conclude with a discussion of the benefits of linking CPS and fluid ability.

### 1.1. Complex Problem Solving (CPS)

Complex problem solving (CPS) is defined in different ways by different authors and researchers, but definitions reflect both the problem-solving and complex aspects of the construct. The lay definition of problem solving is obviously very broad (“the process or act of finding a solution to a problem”) [37], broad enough to describe much of cognition. However, in psychology, a more technical and narrow definition has evolved. In fact, there appear to be two intellectual traditions, one concerning problem solving generally, and the other concerning a particular offshoot that has assumed the name, complex problem solving (CPS). This offshoot, which might be called the German tradition^1^, was initiated by Dörner and colleagues [38], and developed through the sustained efforts of Funke [39] and others. We believe that the general concept of problem solving has been primarily responsible for its inclusion in PISA [40] and PIAAC [25], in employer surveys [7,41], and in occupational task surveys, such as O*NET [6] or the German BIBB Employment Survey [42]. There is a general interest by policy makers and the general public in students’ and employees’ problem solving skills as that concept is generally understood [41]. 

However, problem solving’s narrower, more specific interpretation in the CPS literature following the German tradition is responsible for the inclusion of particular forms of assessments, in PISA 2012, due to that interpretation being responsible for generating a significant research base. We argue here that the two traditions do not necessarily define problem solving in the same way, and the diverging definitions generally result in different problem solving measures. Whether the diverging measures can best be thought of as alternative measures for the same general construct, or whether there is a suggestion of different constructs is an open question. 

#### 1.1.1. General Tradition

A way to appreciate the general tradition on problem solving is by inspecting cognitive psychology textbooks and observing the topics covered in problem solving chapters. Anderson’s [43] textbook includes separate chapters on “Problem Solving” and “Reasoning and Decision Making.” The latter includes the topics of conditional reasoning, quantifiers, inductive reasoning (including Bayes theorem), and decision making (including framing). The Problem Solving chapter is based on the distinction between declarative and procedural knowledge, and it presents problem solving as its essence (“all cognitive activities are fundamentally problem solving in nature;” ([43], p. 237). Importantly, Anderson [43] builds the chapter around what he refers to as the Newell and Simon [44] model of problem solving, which combines thinking in cognitive psychology and artificial intelligence, and which can be characterized as “search through a state space defined by operators” ([43], p. 270). Anderson identifies the three essential features of problem solving: (a) goal directedness; (b) subgoal decomposition; and (c) operator application. Anderson covers the topics of the importance of representation, how operators are acquired (by discovery, instruction, and examples), the use of analogy, and heuristics (difference-reduction, mean-ends analysis), and illustrates the concepts in the context of classical problems such as functional fixedness [45,46] and the two-string problem, set effects and the water jug problem [47] and incubation effects and insight problems. We will later see how the Newell and Simon approach Anderson describes is the basis for information-processing analyses of the problem-solving steps involved in the solution of problems such as number series and figural matrix items found on fluid ability tests.

Another intellectual influence on the general tradition of problem solving follows from Polya’s [48] practical techniques for approaching mathematical problems. He suggested a method of: first, (a) understanding the problem; then, (b) devising a plan; (c) carrying out the plan; and (d) reviewing or reflecting on what was done. He also introduced the concept of using heuristics, such as analogy, generalization, induction, specialization, solving a simpler problem, consider special cases, and working backward, in carrying out the steps. This work has had considerable influence in practical approaches for solving problems [49,50,51]. For example, Bransford and Stein [49] adopted a variant on Polya [48] using the acronym IDEAL (Identify, Define, Explore, Act, Look). Polya’s framework also was the basis for problem solving in PISA 2003 [3], which identified the problem solving processes of understanding, characterizing, representing, solving, reflecting and communicating, represented in the three problem types of decision-making, system analysis and design, and trouble-shooting. 

There are several examples of admissions and selection tests that arguably have been influenced by this general tradition of problem solving. One example pilot tested at the University of Michigan [52] involved the development of a set of Case Scenario Problems treated as potential admissions tests for graduate business school. An example was the personnel shortage problem that asked test takers to imagine that “You are the human resource manager of a manufacturing plant facing a personnel shortage. Your employees are working excessive amounts of overtime and morale is low.” Test takers then were provided a set of materials including current employment figures and job-satisfaction survey results, and asked to answer a set of questions involving problem identification and rationale, solution identification and rationale, information processing, and outcome monitoring and obstacle recognition. Responses were evaluated by alumni and other students. Test takers also were provided with a set of situational judgment tests, referred to as tacit knowledge tests, which described problem scenarios, and were asked to judge the effectiveness (on a seven-point scale) of various solutions to those problems. The study provided some evidence that the two problem solving measures added to standardized tests (i.e., the GMAT) in predicting first year grades, overall grade-point-average, and other outcomes.

An example from industry is McKinsey & Company’s [53] Problem Solving Test, which is administered as a screener to applicants for the business analyst position. Like the Michigan [52] measures, this test describes a series of business scenarios, and assess one’s ability “to solve business problems using deductive, inductive, and quantitative reasoning” [52].

#### 1.1.2. German Tradition

The German tradition also has its origins in the classic problem solving literature. However, the German approach is understood to have taken on its unique identity in the past thirty years [54], perhaps beginning with the work of Dörner [38] who proposed studying CPS through computer-simulated scenarios. An example task is the Tailorshop [39,55,56], a microworld developed in the early 1980s, but used in many research projects in Germany since then (many of the articles about it are in German). Tailorshop is a simulation of a shirt factory that requires the test taker to manipulate a number of variables (e.g., price per shirt, number of shop employees, and wages), which in turn affect the outcomes of a number of other variables (e.g., sales, production rate and employee motivation), at monthly intervals, with the goal being after a year to improve the shop’s total assets, profitability, reputation, and perhaps other factors, which the test taker explores through discovery learning. 

Tailorshop, with 24 variables, is probably the most commonly used in CPS research, but is one of many including Powerplant, LEARN, Spaceshuttle [57], Moro [58], Genetics Lab [59], Water Purification Plant (WPP), Firechief [60], Firefighting (FEUER) [61], and Air Traffic Control [62]. Beyond the German CPS tradition, there are categories of tasks referred to as microworlds, which date back to Pappert and the Logo language [63], game-based assessments [64,65,66], such as Space Fortress [67], complex dynamic control tasks [68]; even intelligent tutoring systems may be thought to fit into this category [69]. Dynamic Decision Making [70] and Systems Thinking have been described as constructs that “overlap greatly with CPS in their respective definitions” ([15], p. 37).

According to Funke [39,54], there are several features common to Tailorshop and other CPS tasks that differentiate them from other tasks such as classical problem solving tasks (e.g., Tower of Hanoi) and cognitive ability tests (e.g., fluid ability tasks). These are that in CPS tasks:

1. there are many variables;
2. which are interconnected;
3. there is a dynamic quality in that the variables change as the test taker interacts with the system;
4. the structure and dynamics of the variables are not disclosed, the test taker must discover them; and
5. the goals of interacting with the system must be discovered.

This last feature does not seem to be universally agreed upon, however. The most central features of those listed seem to be that there is a set of variables whose structure and dynamics must be learned for a test taker to perform well on the task. The relationships between those variables can often be expressed as a set of linear equations [71] linking the output values (**Y**) to input values (**X**), **Y**^t+1^ = **Y**^t^**α** + **Xβ**, where an output (e.g., *Y*_1_) can serve as an input to another output (e.g., *Y*_2_), providing the interrelatedness feature, and the values of **Y**^t^ at a particular moment serve as inputs to the values **Y**^t+1^, for the next time the system is updated, giving the system a dynamic character (there are other models [72] and discussions of their role [73]).

There is a dispute also about the number of variables, as well as time lags, nature of their relationships, and other features, needed to classify a task as complex problem solving. Funke [74] distinguishes between CPS, requiring many variables, and minimal complex systems (MCS) requiring only a few. MCS systems are what have been used in large scale assessments, such as PISA, due to the respondent burden imposed by CPS systems. Funke also argues that the two types of systems are fundamentally different in that, whereas the equations underlying MSC can be taught and learned in a few minutes, CPS tasks are too complex to learn; successful CPS performance is only possible using background knowledge and heuristics. Greiff and Martin [75] argue that CPS is not a task type, but a construct involved in the performance of both complex and minimally complex systems; they argue that the difference is that complex systems additionally tap into knowledge of the particular system being modeled, such as how companies, like Tailorshop, work. This provides a justification for why MCSs are administered in surveys like PISA. The authors agree on the distinctions between the two systems, but perhaps disagree on whether the complexity of many variable systems draws out any particular skill or knowledge that cannot otherwise be accounted for by well-known constructs such as fluid ability and domain knowledge. 

Various studies have been conducted to evaluate the psychometric quality of CPS (and MCS) tasks. A general performance factor across three CPS tasks was found to be predicted by a general cognitive factor [56], although a knowledge factor had a higher correlation. Several studies have found positive correlations, ranging from moderate to fairly high between cognitive ability tests and performance on CPS tasks [60,76,77,78]. A meta-analysis [15], based on 60 samples and over 13,000 participants, estimated a correlation of 0.43 between intelligence and CPS, with the form of the CPS moderating the relationship (e.g., MCS had higher correlations with intelligence, 0.59 vs. 0.47, vs. 0.34, for MSC, LSE, and many-variable-CPS tasks, respectively). 

Greiff et al. [57] found that although cognitive ability was a stronger predictor of student grades, CPS added somewhat to the prediction. Finally, Goode and Beckman [77] found that knowledge of the structural relationships and dynamics (**Y** = **Xβ**), which they manipulated, was an important contributor to performance, along with general cognitive ability needed to apply this knowledge. When structural knowledge was provided the relationship between fluid ability and performance was high; but when not provided, the relationship was low. They suggested that it was unlikely that students acquire structural knowledge simply by interacting with the system. Their study is consistent with other findings that differentiate acquisition and application components to performance [78], and perhaps explains why some CPS studies have found correlations between fluid ability and CPS performance, and others have not [79]: the rule discovery phase, perhaps depending on how it is presented, can tap constructs, such as motivation, unrelated to fluid ability. Whether this component of task performance is important to problem solving generally, or is a task-specific, construct-irrelevant feature is a contested issue, but one difficult to resolve. More generally, the influence of factors other than cognitive ability on cognitive tasks is a problem not limited to CPS [80]. 

### 1.2. Fluid Ability (Gf)

The concept of fluid ability (Gf) is an empirical finding that performance on a wide variety of cognitive tests tends to correlate highly across individuals. The nature of the tests that so correlate, and the regularity of the empirical finding led to Cattell’s [81] assertion that “adult mental capacity is of two kinds, the chief characteristic of which may be best connoted by the use of the terms ‘fluid’ and ‘crystallized’” and that “Fluid ability has the character of a purely general ability to discriminate and perceive relations between any fundaments, new and old” (p. 178). Carroll’s meta-analysis of 450 ability datasets [10] supported the finding of a general fluid ability factor, and suggested that two classes of tasks were the best measures of fluid ability—inductive and deductive reasoning. These were described as follows:

*Induction*: “This factor operates in tasks or tests that present subjects with materials that are governed by one or more implicit rules, or that exhibit or illustrate certain similarities or contrasts. The subject’s task is to discover the rules that govern the materials or similarities and contrasts on which the rules can be based, and then to demonstrate the discovery in some way, either by stating rules or relevant stimulus attributes, or by making appropriate choices among alternatives that are presented” ([10], p. 245, boldface ours). He also concludes that the best and most characteristic tests are “classification, concept formation, correlate completion, induction, letter grouping, letter series, letter sets, letter triangle, locations, marks, matrices, patterns, series, similarities, and verbal classification”. 

*Deductive or Sequential Reasoning*: “This factor operates in tasks or tests that require subjects to start from stated premises, rules, or conditions, and engage in one or more steps of reasoning to reach a conclusion that properly and logically follows from the given premises” ([10], p. 245, boldface ours). Among the most characteristic tests of this factor are deductive reasoning, nonsense syllogisms, inferences, logical reasoning, proverbs, syllogistic reasoning, symbol manipulation, and verbal reasoning. 

Research suggests that fluid ability (Gf) and general cognitive ability (*g*) are essentially the same ability [9,33,34] or very highly correlated [35]. Numerous researchers have found that working memory capacity is either identical to Gf, or very highly correlated [18,19] (a meta-analysis showing a relationship of only rho = 0.63 [82] demonstrates that in any particular study that examines the correlation between a working memory and Gf measure there is considerable task-specific variance attenuating the correlation between the two measures). Wilhelm [83] showed that content similarity or difference can also moderate the relationship between fluid ability and working memory. There is also a suggestion that the correlation between Gf and working memory is higher when Gf is tested under time pressure [84].

Some of the best measures of fluid ability are figural matrices tests and number series tests. Raven’s progressive matrices is widely taken to be the best measure of fluid ability [85,86] and consequently it often is administered in studies as the only fluid ability measure, although this is not an advisable practice [87]. Series tasks have also been found to be among the best measures of fluid intelligence [10]. Detailed information processing analyses have been conducted on these measures and it is instructive to review these.

Information processing models for series tasks (e.g., 245356_; bdcedf_) suggest four stages: relations detection (determining the relationship between contiguous elements), discovery of periodicity (finding the length of the period within the series), completion of the pattern description within the period (identifying relations between elements) and extrapolation, or applying the rules to get an answer [88,89,90]. A finding has been that working memory load is the primary contributor to item difficulty [91,92]. Typically, there are a fairly small number of possible transformations (rules) and only a few to keep track of at a time.

There have been several information-processing analyses of progressive matrices tests [85,93,94,95]. The information processing steps associated with these tests include: (a) finding correspondences between elements across columns, or rows; (b) comparing adjacent corresponding elements; and (c) inducing the element transformation rules. A finding in this research has been that increasing the number of elements or rules necessary to keep track of in working memory increases item difficulty. Typically, there are up to five rules manipulated on up to four elements. 

### 1.3. Formative vs. Reflective Latent Variable Constructs

One difference between the Complex Problem Solving (CPS) and Fluid ability (Gf) constructs may be not so much in the content as in the nature of their definitions. In psychometrics, constructs can be defined in two different ways: in either a formative or reflective measurement model [26,30,96,97]. A reflective measurement model is one in which the construct is assumed to cause variation in the indicators (i.e., test scores, or item responses). This is the framework presumed in classical test theory, item response theory, and factor-analytic ability models, which define abilities as latent variables causing (or underlying) test scores or responses on tests. Abilities are identified through correlations among those scores or responses. Abilities models, as commonly understood, are reflective latent variable models. Gf emerged as a reflective latent variable construct to explain extensive empirical demonstrations of high covariation among a varied set of tasks ranging from inductive to deductive and quantitative reasoning tasks [10]. A characteristic of reflective latent variable constructs is the principle of “indifference of the indicators,” [28] the idea that the same factor will be identified regardless of the particular items or test scores used to measure the factor (this is not the case with formative constructs [30,98]). 

In contrast, formative latent variable constructs are ones in which responses on tests are presumed to be the cause, and not the effect, of the construct. That is, the construct is defined by its constituents, its indicators. The constituents are identified through a process other than accounting for covariation among responses. The process may be a group consensus on what the constituents of a construct might be, or there may be an empirical foundation. An example of the former is the construct of socio-economic status, which by convention is defined as a weighted sum of family income, educational level, and occupational status [97]. An example of the latter would be the stress index [99], which is a checklist of life events (e.g., pregnancy, death of a family member, loss of a job, moving out of one’s residence), which are not necessarily correlated, but together are known to produce emotional stress, which in turn is likely to have effects on outcomes such as physical and psychological health. To some extent, achievement constructs, such as mathematics achievement, can be thought of as formative latent variable constructs as the constituents of the construct are determined from processes such as expert panel meetings. The result is a framework or set of test specifications that identify the processes (e.g., formulating, employing, and interpreting mathematical situations and concepts) and the content (e.g., change and relationships, space and shape, quantity, and uncertainty and data) to be measured ([100], Tables 1.1 and 1.2, p. 38; discussion, pp. 24–45). The construct (e.g., mathematics achievement) is thus defined by the framework and specifications, not by the covariation of any test or item responses. “Indifference of the indicators” does not apply; rather, the construct is specifically defined by which indicators are included. Full construct representation thus is expressed in an assessment by including measures of all the constituents decided upon in the framework or test specifications. This approach is supported in the Test Standards [101] to ensure the avoidance of the validity threat of construct underrepresentation, “the degree to which a test fails to capture important aspects of the construct” (p. 12).

In formative measurement models, indicators are aggregated to form the formative construct or composite. There is a question of how the components are weighted in forming the composite. There are several ways to do so. One is to assign arbitrary weights to the components, such as with sum scores or unit weights. This is a common strategy for many real world indexes, such as the stress index, and the cost-of-living index. Another is to assign weights in proportion to the components’ correlations with the other components, which is what component scores from a principal components analysis are. This is essentially the strategy PISA uses in computing the index of economic, social, and cultural status (ESCS), a socioeconomic status scale [102]. A third method is to include at least two additional outcome variables in the model, a framework known as the Multiple-Indicator-Multiple-Cause (MIMIC) model [96,103]. Weights are assigned to the components in forming a composite that best predicts the two (or more) outcomes (if there is only one outcome then the weights are the same as the regression weights from regressing the outcome on the components, and the composite variable is superfluous).

In many ways, Complex Problem Solving (CPS) as a construct has adhered more closely to the definition of a formative latent variable construct than to a reflective latent variable construct. CPS did not emerge out of factor analytic investigations to explain test score interrelationships, as Gf did. In fact, Carroll [10] does not distinguish reasoning from problem solving (he uses the terms essentially interchangeably, following traditional practice in the field of differential psychology), and he found no factor that might be considered complex problem solving. Instead, CPS developed initially simply as a label to classify a diverse set of tasks used in cognitive psychology (referred to above as the general tradition), and later as a label for a more specific set of tasks that follow certain rules (referred to above as the German tradition). 

Despite its origins, it is possible and appropriate to treat CPS as a reflective construct and evaluate it as such, for example, to evaluate its reliability and dimensionality. Arguably, much of the debate in the literature, although not explicitly recognized as such, appears to be based on whether the construct is treated as a formative or a reflective latent variable construct. 

### 1.4. Conceptual Overlap between CPS and Fluid Ability

Given the definitions of fluid ability provided here along with the exemplar tasks, and the information processing analyses associated with those tasks, it is useful to compare process descriptions of fluid ability and CPS tasks. Both kinds of tasks are broadly consistent with Carroll’s [10] definition of induction and deductive reasoning tasks (see above). In addition, both CPS and fluid ability tasks require tracking several interconnected variables (or elements or rules) (CPS features 1 and 2), and the relationships among them are typically not disclosed (CPS feature 4), at least with inductive reasoning tasks. CPS tasks differ from fluid ability tasks in that there is not a dynamic quality to fluid ability tasks (CPS feature 3), and the goal for fluid ability tasks is always clear, whereas it is not always clear with CPS tasks (CPS feature 5). 

However, as noted above, it is disputed whether feature 5 is a defining feature of CPS tasks. A defining feature of problem solving is its goal directedness. However, if two participants working on the same task have different goals, which can be the case in some CPS tasks, it is not clear how a particular score based on task performance can be interpreted. Choice of goals may reflect individual values or subjective incentives, but performance on a task for which goals are not clearly provided by the context or the task administrator cannot be considered a measure of problem solving skill.

This leaves possibly two features differentiating CPS and Gf tasks. One concerns the dynamic quality of CPS tasks. It has not been clearly shown that this particular feature is more than an incidental task feature, using Irvine’s terminology. An argument could be made that the dynamic quality of CPS tasks invokes a test-operate-test-exit (TOTE) cycle [104] and that a similar TOTE approach is taken in the solution of, for example, progressive matrices and series problems. In these, elements are identified, operators applied to them, the results are evaluated, and a determination is made about whether the correct rule has been identified or not. The qualitative nature of the processing in the two tasks is thus not fundamentally different; it is simply the case that computer administration affords dynamic updating which can be thought of essentially as a record keeping of the results of TOTE cycles, which otherwise might have to be retained in working memory. 

The other differentiating feature is the number of rules and elements that have to be attended to. The importance of this differentiating feature to the identity of a CPS task is the subject of current debate (e.g., [74,75,77,105]), and certainly is not a settled issue at this point. It is reasonable to assume that many-variable CPS tasks invoke domain knowledge more so than MCS tasks do, and therefore are more likely to be multidimensional. 

### 1.5. Relationship between CPS and Fluid Ability in the World of Work

The previous section suggested that there is a good deal of conceptual overlap between the constructs of fluid ability and complex problem solving, but there may also be some differentiating features. A program of research could and should be undertaken to identify convergent and discriminating validity evidence for a CPS factor alongside related factors such as fluid ability, general problem solving, and minimal complex systems (MCS) factors. This would be useful for clarifying the nature of problem solving constructs, and the role of problem solving in school and in the workplace. 

However, while such a program of research is underway, it is useful to note that there is already data on the importance and the level of complex problem solving skill and fluid ability, as well as domain-specific knowledge needed for the performance of hundreds of occupations in the USA Analysis of the importance of these three knowledge, skill, and ability areas across jobs should shed light on the some of the issues addressed in the current CPS literature including: (a) the relationship between CPS and fluid ability; (b) the relative importance of the value of CPS skill and fluid ability in the labor market; and (c) whether there is evidence that CPS skill includes features beyond those captured by fluid ability that are valued in the workplace. We also look at the relative role of domain-specific knowledge as an additional potentially important factor.

The Occupational Information Network (O*NET) is a database maintained and frequently updated by the USA Department of Labor since 1998, which contains (among other items) ratings on factors such as skills requirements and work activities for jobs clustered into 1102 occupations, such as Lawyers, Biochemical Engineers, Police Detectives, and Travel Agents [6,36,106,107]. The Ratings are provided by job incumbents, occupational experts, or occupational analysts, depending on the rating, and they are organized into six broad domains (e.g., worker characteristics, worker requirements, experience requirements, occupational requirements, workforce characteristics, and occupation-specific information).^2^ Approximately 177 ratings are collected altogether for each occupation (52 abilities (23 cognitive), 35 skills, 33 knowledge areas, 41 generalized work activities, and 16 work styles), in addition to a six-item education and training questionnaire, and a 15-item background questionnaire.

For this study we focus on three sets of ratings:

(a) the 23 cognitive abilities in the worker characteristics domain (items 1 to 23 on the O*NET Abilities Questionnaire) (see Table 1);
(b) the 33 knowledge areas in the worker requirements domain (items 1 to 33 on the O*NET Knowledge Questionnaire); and
(c) a single Complex Problem Solving (CPS) rating in the cross-functional skills set within the worker requirements domain (item 17 on the O*NET Skills Questionnaire).^3^

For cognitive abilities, we use a principal component score from the importance ratings of 23 abilities as our measure of fluid ability. For the knowledge measure, we use the highest importance rating from the 33 knowledge areas as our measure of the importance of domain-specific knowledge for that occupation.

The questions we address with these data are: (a) What is the relationship between fluid ability and complex problem solving skill? (b) How relatively valuable are these skills based on the correlation between the importance of these skills in different occupations and the average wages paid in those occupations? (c) Do these general skills predict wages when controlling for knowledge? 

## 2. Materials and Methods

**Figure 1:**
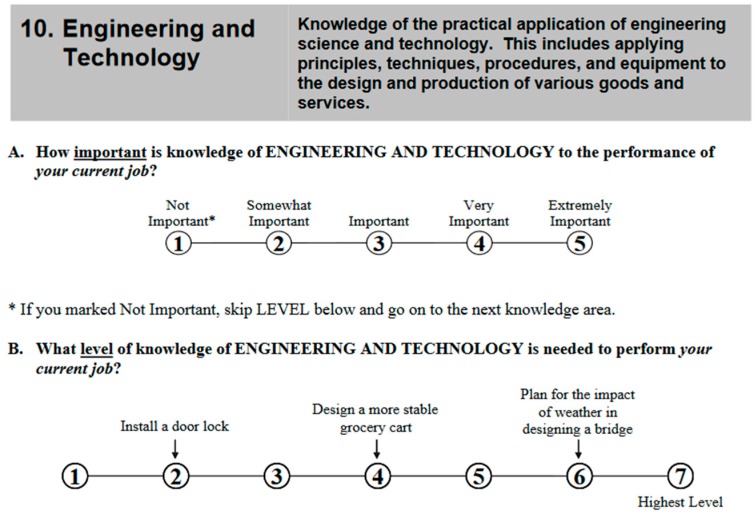
Sample knowledge item from the O*NET Knowledge Questionnaire.

Ratings for the three areas (cognitive abilities, knowledge areas, complex problem solving) were conducted slightly differently. For the knowledge areas, ratings were provided by job incumbents who were selected through a two-stage random sampling in which businesses employing workers in the targeted occupation are identified then incumbents within those occupations at particular businesses are randomly chosen. Raters were provided with training materials and were compensated for completing the surveys, which took about 30 min to complete. The ratings we analyzed are averages across anywhere between 15 and 200 job incumbents, depending on the occupation. Figure 1 shows an example of an item from the knowledge questionnaire (Appendix A, Table A1 lists the 33 knowledge items).

For cognitive abilities and Complex Problem Solving (CPS) skills, ratings were provided by eight occupational analysts [108]^4^, who received extensive training on the occupations, the definitions of skills, and the ratings process. Analysts had at least two years of work experience, had completed two years of graduate education in organizational psychology, industrial relations, or human resources, and had completed courses in job analysis and methods. 

**Figure 2:**
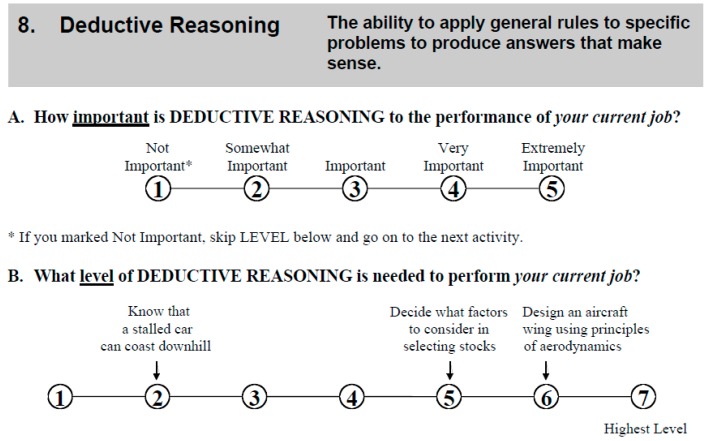
Sample item from the O*NET Abilities Questionnaire.

Figure 2 shows the Deductive Reasoning item from the Abilities Questionnaire. The Inductive Reasoning Item (not shown) is given the definition “The ability to combine pieces of information to form general rules or conclusions (includes finding a relationship among seemingly unrelated events).” It also provides the level anchors “decide what to wear based on the weather report” (level 2), “Determine the prime suspect base on crime scene evidence” (level 4), and “Diagnose a disease using results of many different lab tests” (level 6). (Table 1 lists all the cognitive abilities rated.)

**Figure 3:**
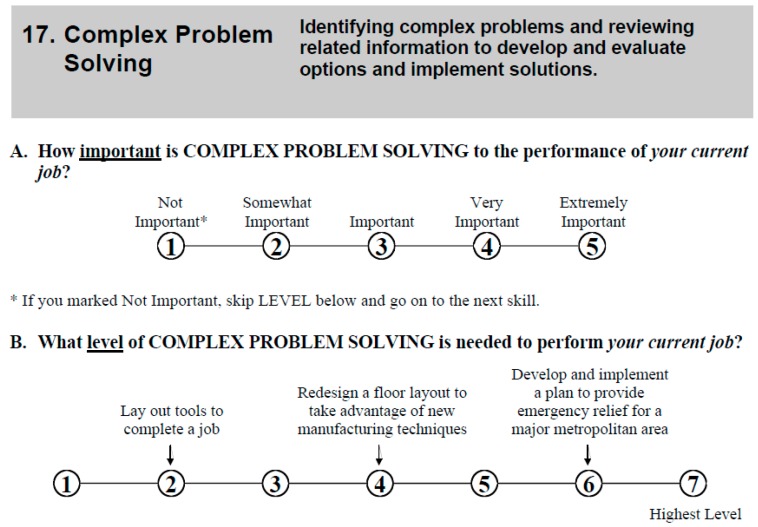
Complex Problem Solving item from the O*NET Skills Questionnaire.

Figure 3 shows Complex Problem Solving Item from the Skills Questionnaire. Note the level anchors: an example Level 4 Complex Problem Solving job would be to “Redesign a floor layout to take advantage of new manufacturing techniques,” and a Level 6 job would be to “Develop and implement a plan to provide emergency relief for a major metropolitan area.” Appendix A, Table A2 lists all the items from the Skills Questionnaire; CPS is Item 17.

For each occupation, analysts provided mean importance ratings (1–5) and mean level ratings (1–7) using the scale shown in Figure 2 and Figure 3, which included the level anchors. For reporting, these 1–5 and 1–7 observed ratings (X) are converted to a 0–100 scale by Y = (X − 1)/(highest possible rating − 1) × 100.

Importance and level ratings have been found to be nearly indistinguishable, with a correlation estimated as 0.96 in one study ([109], p. 6). For our purposes, we only analyzed importance ratings. Reliability analyses show that even the single ratings (e.g., CPS) have high reliability (ICC, based on eight raters = 0.83 (importance), 0.90 (level)) [86]. All data analyzed for this study were obtained from the O*NET Online [6].

A potential limitation of the results of the study is that our measure of CPS is based on one item, albeit averaged over eight raters. However, the justification for using one rating is that the one rating is a direct measure of the importance of complex problem solving for various occupations as determined by occupational analyst experts.

## 3. Results

First, a principal components analysis was conducted on the mean importance ratings (across the eight raters) of the 23 abilities variables from the Abilities Questionnaire. Three components had eigenvalues greater than 1. Table 1 presents the loadings for the first three components from the analysis.^5^ The two highest loadings were for Deductive Reasoning and Inductive Reasoning, which is consistent with an interpretation of the first component as a Gf factor. We refer to it as *g*/Gf throughout the remainder of the paper. The other two components appear to reflect a spatial vs. non-spatial distinction (Component 2), and a number factor (Component 3).

### 3.1. Correlations among Skills

Next, we computed the correlation between the three sets of scores, the *g*/Gf component, the Complex Problem Solving importance rating, and Knowledge importance ratings. The results are shown in Table 2. Note that *g*/Gf and CPS are very highly correlated (*r* = 0.86). Although this correlation is higher than correlations between *g*/Gf measures and CPS measures found in meta-analyses of test performance across individual test takers [15], note that our estimate is not strictly comparable to those estimates and is likely higher due to common: (a) methods (ratings on a common scale under similar conditions); (b) raters; and (c) occasions, and the fact that it is a group- (occupation) rather than individual-level correlation [111]. 

### 3.2. Overall Regression Analysis of Occupation Median Wages

Next, we computed a series of regression models with occupation log median wages serving as the dependent measure and the three skills factors as the predictors. Model 1 includes only *g*/Gf as a predictor; Model 2 adds CPS; Model 3 includes *g*/Gf and Knowledge; and Model 4 includes all three predictor variables. Table 3 presents the findings. 

Comparing Model 2 to Model 1 shows that CPS, despite being a single item measure, adds significantly to *g*/Gf in predicting wages, *F* (1, 688) = 16.6, *p* < 0.01. Comparing Model 4 to Model 3 shows that this holds even when controlling for knowledge, *F* (1, 688) = 18.6, *p* < 0.01.

### 3.3. Within Job Zone Regression Analyses of Log Median Wages

O*NET occupations are clustered into five job zones that represent the general level of preparation, education, and experience that a job requires. Job Zone 1 occupations require little previous work experience and some require a high school education or equivalent (e.g., cashiers, clerks, and dishwashers). Job Zone 2 occupations require a high school diploma and some previous work experience (e.g., customer service representatives, security guards, and bank tellers). Job Zone 3 occupations typically require vocational school training, on-the-job experience or an associate’s (two-year) degree (e.g., electricians, barbers, and medical assistants). Job Zone 4 occupations typically require a bachelor’s degree, and several years of work experience (e.g., accountants and sales managers). Job Zone 5 occupations typically require a master’s or doctoral degree and five or more years’ experience (e.g., librarians, lawyers, biologists, and surgeons).

We conducted regressions similar to those shown in Table 3 (overall), separately by Job Zone. We ignored Zone 1 because the *N* was too small. Table 4, Table 5, Table 6 and Table 7 present the results.

For Job Zones 2 and 3 (Table 4 and Table 5), the results qualitatively match the overall results in that CPS adds significantly to *g*/Gf in predicting wages, even when controlling for knowledge. For the Model 2 vs. Model 1 comparisons, *F* (1, 248) = 16.6 (Zone 2), and *F* (1, 171) = 3.9 (Zone 3). For the Model 4 vs. Model 3 comparisons, *F* (1, 248) = 15.8 (Zone 2), and *F* (1, 171) = 4.2 (Zone 3), all significant at *p* < 0.01. However, for Job Zones 4 and 5, none of the model *R*^2^ are significantly different from zero. This may indicate that there is a curvilinear relationship between skills ratings and wages at the higher end of the experience and education levels (i.e., in Job Zones 4 and 5). This could be explained *g*/Gf and CPS being important for getting incumbents qualified for jobs at the higher level occupations, even if wages are not related to those factors within job zones. It could be that higher education screens incumbents for *g*/Gf and CPS skill. This does not invalidate the conclusions of a positive relationship between skill and wages; it simply suggests that the relationship may be nonlinear.

## 4. Discussion

The purpose of this study was to examine the relationship between complex problem solving and general fluid ability both conceptually and empirically. We conducted conceptual comparisons by examining researchers’ definitions of the two factors, prototypical tasks, and the information processing analyses of performance on those tasks. We also proposed considering the distinction between formative and reflective measurement models that might identify the constructs. 

In the case of fluid ability, there is a well-established line of research that has led to fairly clear definitions of the construct, a notion that this is a reflective measurement construct, prototypical tasks, well defined information-processing models of task performance, a nomothetic span, and knowledge of its development and growth due to age, schooling, and interventions. In the case of CPS, there are several definitions of the construct stemming from various traditions, exemplar tasks within the various traditions, and useful information processing models for those tasks. At a fairly general level, fluid ability and complex problem solving ability certainly overlap conceptually, substantially, if not completely. However, with more specific definitions of CPS, there are conceptual distinctions, perhaps the most important being that CPS may more explicitly include domain-specific knowledge as part of its definition. 

In fact, there are three fairly broad definitions of the CPS construct. The general tradition defines problem solving as a goal-directed search through a problem space using operators (e.g., adding two numbers, comparing two elements), methods (e.g., hill climbing, means-ends analysis), and heuristics (e.g., consider extreme cases, work backwards) acquired through observation, doing, direct instruction, and analogy. Prototypical tasks from this tradition include Tower of Hanoi, Cryptarithmetic [44], the eight-tile problem, many of the problems solving tasks administered in PISA 2003 and 2012 [4], and even arithmetic word problems and situational judgment tasks as in the McKinsey Problem Solving Test. Arguably, this is the common understanding of problem solving, and when reports on students problem solving ability appear (e.g., PISA), or when an employer is asked how much “complex problem solving ability” is required for a particular job, this is the definition likely to be invoked by the respondent. 

The emerging “German tradition” (sometimes referred to as “European tradition”) provides two broad definitions of complex problem solving ability that specialize the general definition somewhat. The initial CPS definition was that it was the ability to succeed in microworlds involving many interconnected variables that test takers could manipulate dynamically to study their structural relationships with one another, and to discover goals to pursue while doing so. This is a clear definition that enables a complexity characterization (e.g., more variables and relationships), and that is amenable to an information-processing analysis of task performance. However, this definition is problematic in providing a basis for classifying a task as measuring a skill for two reasons.

First, letting the user rather than test designer determine the user’s goal means that performance will at least partly reflect goal searching in addition to problem solving. The interpretation of a score then is ambiguous. Second, such systems can be extremely complex, and only solvable through reliance on background knowledge, such as about how a real-world system simulated by the microworld actually works. In this case, CPS is a multidimensional construct of a basic CPS ability dimension combined with knowledge of the particular CPS domain being investigated. For these two reasons, one’s measured CPS ability therefore would not be invariant, but would vary with what goal the user might be pursuing, and with the domain being investigated. An auto mechanic could be considered high in CPS for an automobile CPS task, but low in CPS for a medical diagnosis task and vice versa for a physician. 

As a result of this problem, combined with the respondent burden associated with many-variable CPS systems, a third definition of CPS has emerged recently under the heading of minimal complex system (MCS). An MCS is similar to the many-variable microworlds, but it includes fewer variables (less than 10), provides clear goals, minimizes the reliance on domain knowledge, and is typically administered in a far shorter time (a few minutes). These features make MCS versions of CPS ideally suited for assessment purposes, and for this reason the problem solving tasks administered in PISA 2015 were inspired by this concept [5]. Meta-analyses show that performance in MCSs is more highly correlated with fluid ability than many-variable CPS task performance is. This supports the idea that there might not be a CPS ability per se, but that fluid ability and domain knowledge are determinants of success in CPS tasks, which can vary dramatically by the domain they are situated in.

The empirical findings based on experts’ ratings of the skill requirements in various occupations are consistent with the idea of a strong overlap between fluid ability and general CPS skill. The correlation between the importance of CPS and fluid ability across 691 occupations was found to be 0.86. This correlation is roughly equal to the correlation between performance of problem solving tasks in PISA and school achievement generally [112]. 

We should acknowledge a couple of potential limitations to our interpretation of the findings presented here. First, as noted above, our measure of complex problem solving (CPS) is based on ratings provided by eight raters on a single occasion on a single item (the importance of CPS on job performance, see Figure 3). The fact that the eight raters produce high interrater agreement does not mitigate the validity threat due to relying on a single indicator of the importance of CPS in occupations. Second, we may be viewed as inferring relationships between individual-level abilities (CPS and *g*/Gf) based on an analysis of ability importance ratings gathered at the occupation level, an ecological correlation. While it is a fallacy to assume that ecological correlations hold at the individual level [113], we present the ecological correlation in this study as a supplement to the already established finding that CPS and *g*/Gf are related at the individual level [15]. 

Our study of importance ratings for occupations provides evidence that there may be some ability captured by the CPS construct that is not fully captured by the fluid ability construct. We found that although the correlation between fluid ability and CPS skill was quite high, CPS added to Gf in predicting wages, indicating employers value whatever is unique (from Gf) about CPS skill. If what differentiates CPS from Gf is that CPS reflects the ability to solve problems in a knowledge domain, then it would make sense that employers value that skill above raw intellectual ability. 

Due to the efforts of the German school of problem solving researchers, significant progress has been made in our understanding of complex problem solving, both in substantive findings concerning the nature of human cognition, and in tools for investigating the construct. Future research is likely to continue to explore the importance of domain knowledge as part of the complex problem solving construct.

## Figures and Tables

**Table 1:** Principal components loadings of the 23 cognitive abilities (Mean Importance ratings).

Cognitive Ability	Component 1 (*g/*Gf)	Component 2 (Spatial)	Component 3 (Number)
Deductive Reasoning	0.90	−0.12	0.01
Inductive Reasoning	0.88	−0.12	0.07
Written Comprehension	0.85	−0.37	0.03
Written Expression	0.84	−0.34	0.08
Fluency of Ideas	0.84	−0.14	−0.09
Originality	0.80	−0.15	−0.05
Information Ordering	0.80	0.22	−0.17
Category Flexibility	0.79	0.11	−0.26
Oral Comprehension	0.77	−0.43	0.28
Memorization	0.77	−0.06	0.06
Problem Sensitivity	0.76	0.24	0.26
Oral Expression	0.74	−0.50	0.30
Speed of Closure	0.71	0.42	0.10
Math Reasoning	0.70	0.04	−0.56
Flexibility of Closure	0.64	0.63	0.03
Number Facility	0.65	0.10	−0.57
Selective Attention	0.43	0.53	0.32
Time Sharing	0.49	0.38	0.55
Perceptual Speed	0.33	0.81	−0.03
Visualization	0.17	0.72	−0.24
Spatial Orientation	−0.31	0.66	0.23

**Table 2:** Correlations among the skills variables and occupation log income.

Predictor Variable	*g*/Gf	CPS	Knowledge	Log Median Wages
*g*/Gf	1.00	0.86 *	0.63 *	0.39 *
CPS	-	1.00	0.58 *	0.42 *
Knowledge	-	-	1.00	0.28 *

Notes: *N* = 692 (occupations). *g*/Gf = 1st principal component score; CPS = mean Complex Problem Solving Rating; Knowledge = Highest mean knowledge rating for an occupation. * *p* < 0.001.

**Table 3:** Regression models pooling over job zones (DV: natural log median annual wages, ln).

Predictor Variable	*M (SD)*	Model 1	Model 2	Model 3	Model 4
*g*/Gf	−0.01 (0.99)	0.19 * (0.02)	0.06 (0.01)	0.17 * (0.02)	0.05 (0.03)
CPS	−0.02 (0.99)	-	0.15 * (0.03)	-	0.15 * (0.03)
Knowledge	2.53 (1.03)	-	-	0.04 * (0.02)	0.03 (0.02)
*R*^2^	-	0.15	0.18	0.16	0.18
*SSE*	-	139.10	135.13	138.25	134.60

Notes: *N =* 691 (occupations); *g*/Gf = 1st principal component score; CPS = mean Complex Problem Solving Rating; Knowledge = Highest mean knowledge rating for an occupation (all ratings are scaled 0, 1). Unstandardized regression weights shown (in parentheses, standard errors). ln (median annual wage) *M* = 10.61 (*SD* = 0.49). * *p* < 0.05.

**Table 4:** Regression models for Job Zone 2 (DV: natural log median annual wages, ln).

Predictor Variable	*M (SD)*	Model 1	Model 2	Model 3	Model 4
*g*/Gf	−0.74 (0.67)	0.14 * (0.01)	0.04 (0.03)	0.13 * (0.03)	0.04 (0.03)
CPS	−0.71 (0.62)	-	0.15 * (0.04)	-	0.15 * (0.04)
Knowledge	2.01 (0.89)	-	-	0.02 (0.02)	0.01 (0.02)
*R*^2^	-	0.11	0.16	0.11	0.16
*SSE*	-	17.78	16.67	17.71	16.65

Notes: *N =* 251 (occupations); Natural log median annual wage *M* = 10.42 (*SD* = 0.28). * *p* < 0.05.^6^

**Table 5:** Regression models for Job Zone 3 (DV: natural log median annual wages, ln).

Predictor Variable	*M (SD)*	Model 1	Model 2	Model 3	Model 4
*g*/Gf	0.11 (0.64)	0.16 * (0.01)	0.09 (0.02)	0.15 * (0.04)	0.08 (0.05)
CPS	−0.03 (0.62)	-	0.10 * (0.05)	-	0.10 * (0.05)
Knowledge	2.47 (0.76)	-	-	0.01 (0.03)	0.02 (0.03)
*R*^2^	-	0.11	0.12	0.10	0.13
*SSE*	-	14.79	14.46	14.78	14.43

Notes: *N =* 174 (occupations); ln (median annual wage) *M* = 10.70 (*SD* = 0.31). * *p* < 0.05.

**Table 6:** Regression models for Job Zone 4 (DV: natural log median annual wages, ln).

Predictor Variable	*M (SD)*	Model 1	Model 2	Model 3	Model 4
*g*/Gf	0.84 (0.60)	0.11 (0.09)	0.04 (0.14)	0.11 (0.09)	0.04 (0.14)
CPS	0.79 (0.67)	-	0.09 (0.12)	-	0.09 (0.12)
Knowledge	3.02 (0.86)	-	-	−0.04 (0.07)	−0.04 (0.07)
*R*^2^	-	0.01	0.02	0.02	0.02
*SSE*	-	47.55	47.33	47.41	47.21

Notes: *N =* 124 (occupations). ln (median annual wage) *M* = 10.80 (*SD* = 0.62).

**Table 7:** Regression models for Job Zone 5 (DV: natural log median annual wages, ln).

Predictor Variable	*M (SD)*	Model 1	Model 2	Model 3	Model 4
*g*/Gf	0.97 (0.57)	−0.28 * (0.11)	−0.29 * (0.13)	−0.26 * (0.11)	−0.28 * (0.13)
CPS	1.14 (0.56)	-	0.03 (0.13)	-	0.04 (0.13)
Knowledge	3.49 (0.95)	-	-	0.04 (0.07)	0.04 (0.07)
*R*^2^	-	0.06	0.06	0.06	0.06
*SSE*	-	44.95	44.93	44.83	44.80

Notes: *N =* 106 (occupations); ln (median annual wage) *M* = 10.84 (*SD* = 0.67). * *p* < 0.05.

## Footnotes

1 Although some authors refer to this as the European tradition, it seems that almost all research comes from Germany, and U.K. research seems more in line with the American tradition.

2 Each domain is broken down further. For example, worker characteristics include abilities (defined as “Enduring attributes of the individual that influence performance”), interests, values, and styles (i.e., personality). Worker requirements include basic and cross-functional skills, knowledge, and education. Cross-functional skills include Complex Problem Solving, Time Management, and 30 others. Knowledge includes 33 knowledge areas.

3 In the original O*NET prototype questionnaire, Complex Problem Solving was rated through eight constructs: (a) Problem Identification; (b) Information Gathering; (c) Information Organizing; (d) Synthesis/Reorganization; (e) Idea Generation; (f) Idea Evaluation; (g) Implementation Planning; and (h) Solution Appraisal [83]. In the revised questionnaire, these eight ratings were replaced by a single rating for Complex Problem Solving to reduce rater burden.

4 Skills ratings originally were provided by job incumbents, but more recently have been provided by occupational analysts to avoid problems of incumbent ratings inflation and because of analysts’ understanding of the constructs being rated [84].

5 Our results replicate the findings of Hunt and Madhyastha [110], with some differences. The pattern of loadings on the first principal component was identical across the two analyses, with the only negative loading across both studies being Spatial Orientation. Loadings on the earlier study’s first component were consistently larger than those in our study, which is consistent with the fact that Hunt and Madhyastha accounted for 58% of the variance while our analysis accounted for 50% of the variance in ability ratings. We attribute these differences to the prior study being conducted over five years ago. O*Net ratings are periodically updated; the mixture of the jobs ratings varied somewhat between our and Hunt and Madhyastha’s studies.

6 Note that going from Job Zone 2 to 3, log median annual wages goes from 10.42 to 10.70. Because we are using natural logs, this difference can be interpreted as roughly a 28% wage increase (actually, 32%); similarly going from Zone 4 (10.80) to Zone 5 (10.84) suggests roughly a 4% increase (actually 4%).

## Appendix A

**Table A1:** O*NET Knowledge Questionnaire Items (numbered as in the questionnaire).

1. Administration and Management
2. Clerical
3. Economics and Accounting
4. Sales and Marketing
5. Customer and Personal Service
6. Personnel and Human Resources
7. Production and Processing
8. Food Production
9. Computers and Electronics
10. Engineering and Technology
11. Design
12. Building and Construction
13. Mechanical
14. Mathematics
15. Physics
16. Chemistry
17. Biology
18. Psychology
19. Sociology and Anthropology
20. Geography
21. Medicine and Dentistry
22. Therapy and Counseling
23. Education and Training
24. English Language
25. Foreign Language
26. Fine Arts
27. History and Archeology
28. Philosophy and Theology
29. Public Safety and Security
30. Law and Government
31. Telecommunications
32. Communications and Media
33. Transportation

Note: For each knowledge area, job incumbents were asked the following two questions: (a) How important is knowledge of X (or X knowledge) to the performance of your current job? (b) What level of knowledge of X (or X knowledge) is needed to perform your current job?

**Table A2:** O*NET Skills Questionnaire Items (numbered as in the questionnaire).

1. Reading Comprehension
2. Active Listening
3. Writing
4. Speaking
5. Mathematics
6. Science
7. Critical Thinking
8. Active Listening
9. Learning Strategies
10. Monitoring
11. Social Perceptiveness
12. Coordination
13. Persuasion
14. Negotiation
15. Instructing
16. Service Orientation
17. Complex Problem Solving
18. Operations Analysis
19. Technology Design
20. Equipment Selection
21. Installation
22. Programming
23. Quality Control Analysis
24. Operations Monitoring
25. Operation and Control
26. Equipment Maintenance
27. Troubleshooting
28. Repairing
29. Systems Analysis
30. Systems Evaluation
31. Judgment and Decision Making
32. Time Management
33. Management of Financial Resources
34. Management of Material Resources
35. Management of Personnel Resources
